# Adolf Lorenz and the Lolita Armour Case

**DOI:** 10.1007/s00264-020-04620-y

**Published:** 2020-06-03

**Authors:** Gerold Holzer, Lukas A. Holzer

**Affiliations:** 1 Department of Orthopedics and Trauma Surgery, Medical University of Vienna, Waehringer Guertel 18-20, 1090 Vienna, Austria; 2 AUVA Trauma Center Klagenfurt, Klagenfurt am Woerthersee, Austria

**Keywords:** Adolf Lorenz, Armour family, Congenital dislocation of the hip, Bloodless surgery, 1902

## Abstract

Almost 120 years ago, in 1902, the American multimillionaire J. Ogden Armour invited the Austrian orthopaedic surgeon Adolf Lorenz, professor at the University of Vienna, to treat his daughter Lolita. Lolita was born premature in 1896 and spent the first months of her life in an incubator. Later she was diagnosed with congenital dislocation of both hips. Lorenz had developed a “bloodless” treatment method and was invited by the Armour family to Chicago to “operate” on Lolita. Both hips had already been treated by an American orthopaedic surgeon before but without a satisfactory result. Lorenz should achieve a better one. The operation was performed in Chicago on 12 October 1902 and was accompanied by a very large media spectacle. This article is mainly based on contemporary newspaper reports.

Lolita Armour (Fig. 1), the granddaughter of Philipp D. Armour^1^, one of the richest men in America at the end of the nineteenth century, was born premature (34th week of pregnancy) and weighed only 3 lb. In order to ensure her survival, her grandfather had an incubator built, in which she had to spend her first year. Later, she was diagnosed with congenital dislocation of both hips (CDH). After the grandfather’s death, Lolita’s father became the company’s CEO and increased the family’s fortune to $100 million.^2^

**Fig. 1:**
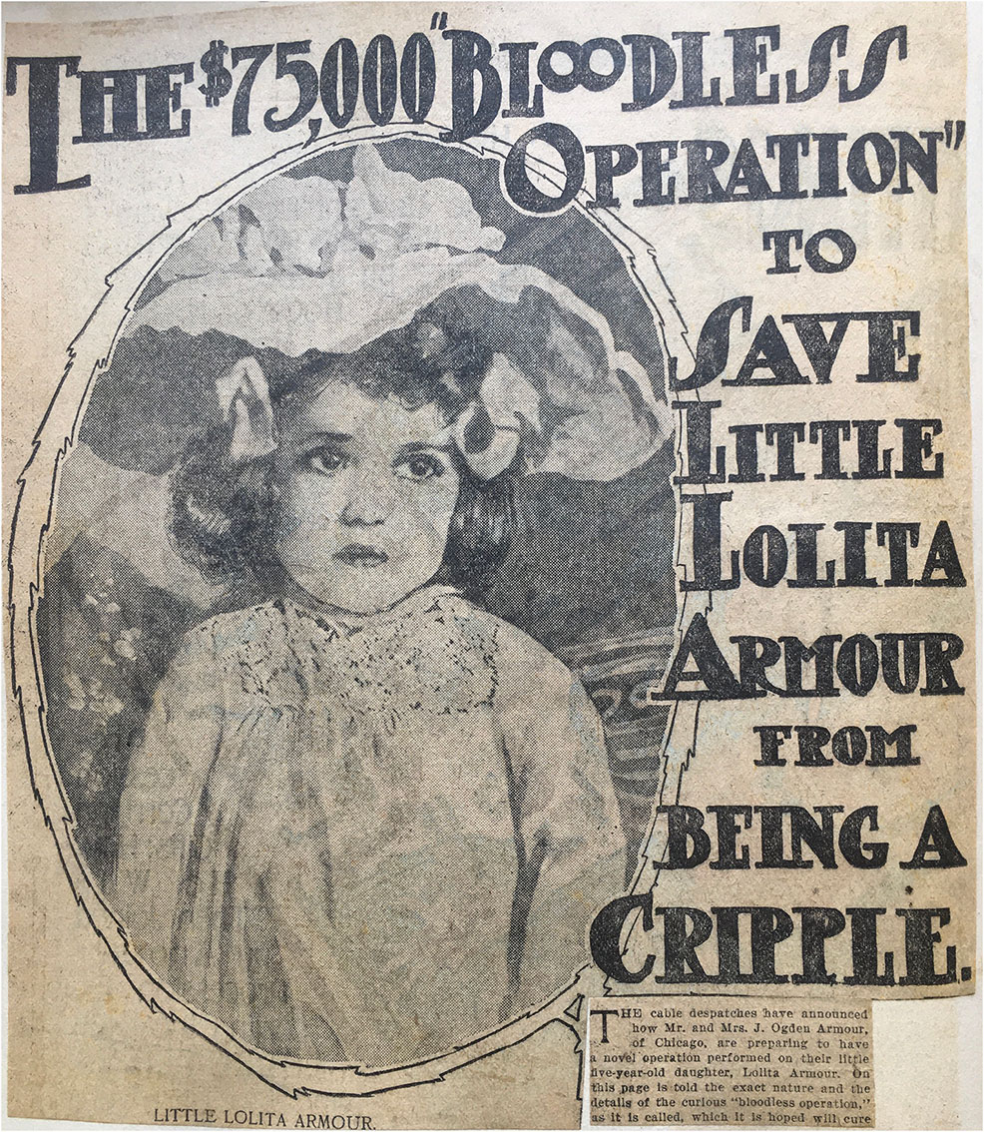
Lolita Armour (at the age of surgery) (Lorenz Archiv Vienna—Photograph: G. Holzer)

Adolf Lorenz (Fig. 2) [1], an Austrian orthopaedic surgeon, was born to poor parents in 1854. His uncle enabled him to study at the gymnasium of the Benedictine Abbey of St. Paul in Lavanttal and later in the Austrian state gymnasium in Klagenfurt. Then he studied medicine at the University of Vienna [2] receiving his MD in 1880. During his training in surgery with Eduard Albert [3], Lorenz [4–8] developed an allergy to carbol. So, Lorenz, among other diseases, also devoted to CDH [9–11] and began to focus on “bloodless surgery” [12].

**Fig. 2:**
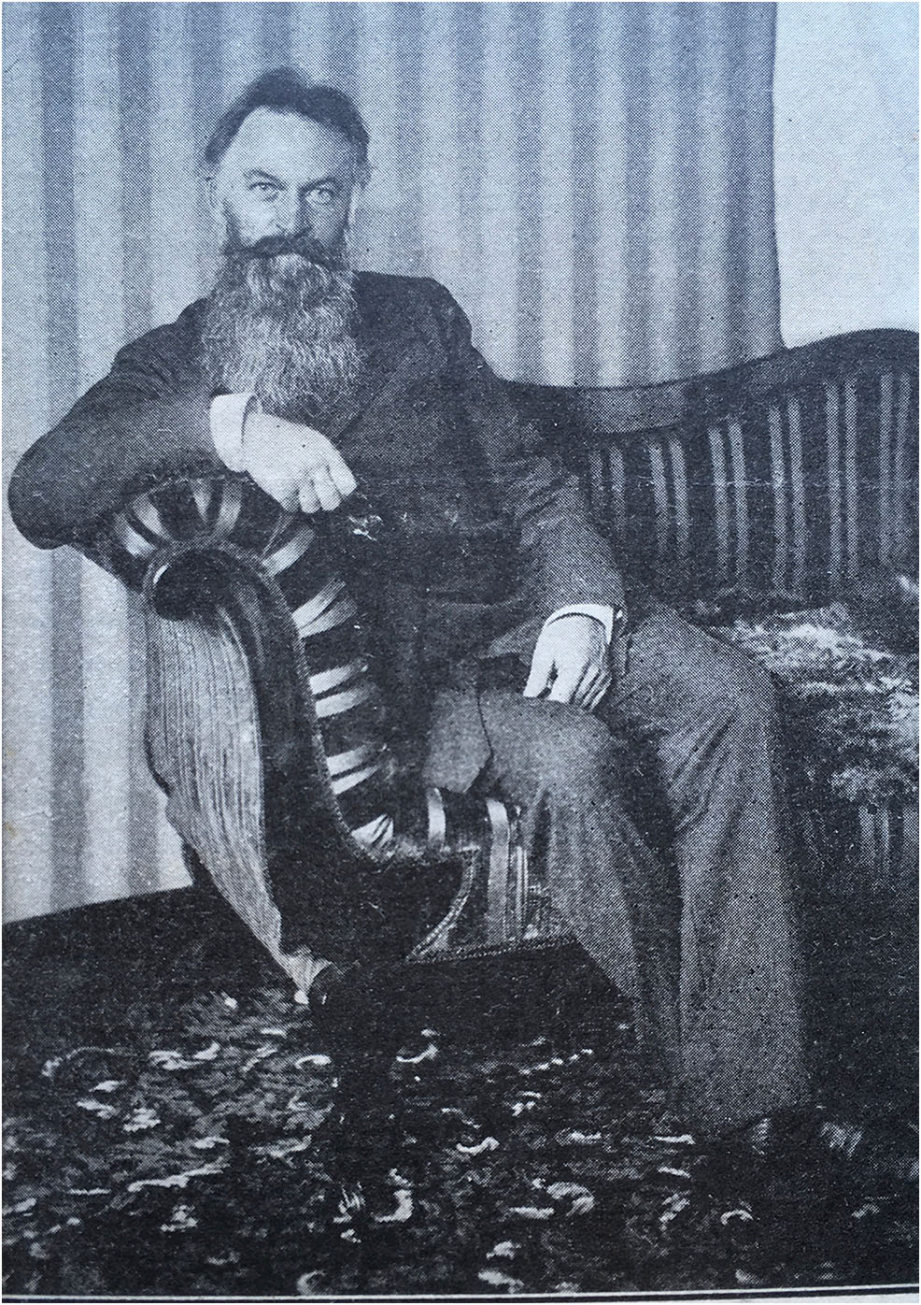
Adolf Lorenz in Chicago 1902 (Lorenz Archiv—Photograph: G. Holzer)

In 1896, Lorenz published the first results,^3^ his book *On the Healing of Congenital Hip Dislocation* was published in 1900 (Fig. 3) [13]. At that time, he had already gained experience from more than 1000 patients^4^ and presented the results at XIII International Congress in Paris.^5^

**Fig. 3:**
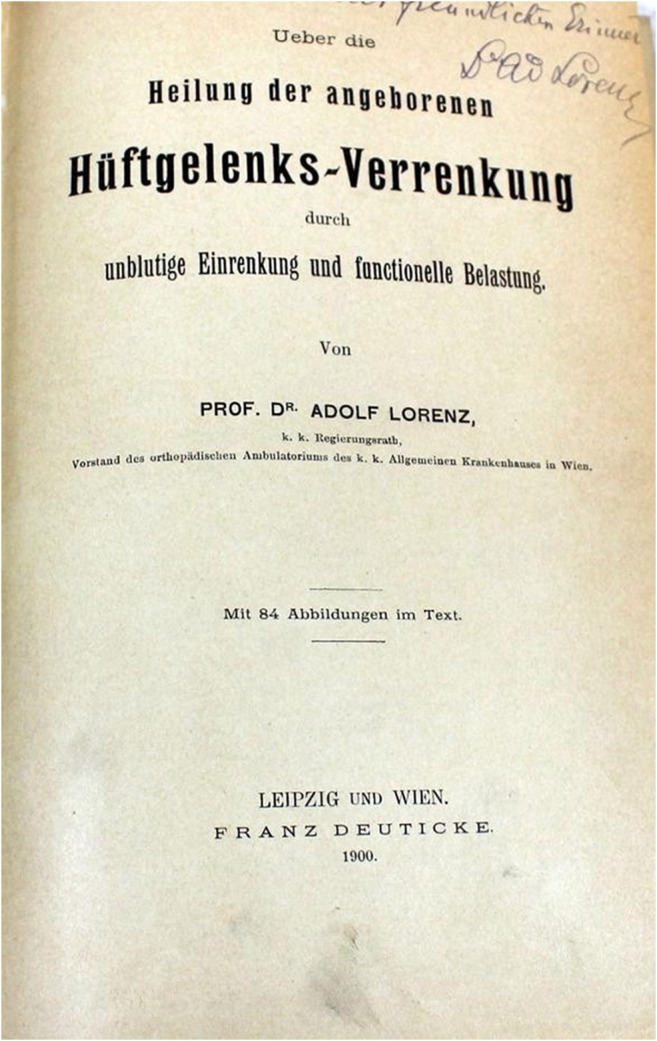
Title page of “On the treatment of the congenital hip dislocation by “unbloody” reposition and functional loading” published 1900 (Photograph: G. Holzer)

American doctors, who were world leaders in orthopaedics at the time, also dealt with CDH [14]. Above all, John Ridlon, already in 1890 focused in a lecture on “The subject of treatment of hip-joint disease is narrowed down to fixation and traction.”^6^

Lorenz’s method was already known in America in 1898. At the meeting of the “New York Academy of Medicine - Section on Orthopedic Surgery” on 18 March 1898, Dr. Royal Whitman reported on a case of CDH cured by Lorenz method of forcible reduction.^7^ In 1899, John Ridlon of Chicago commented on the “Mechanical Treatment of Hip Joint Disease.”^8^

In October 1900, Dr. John Ridlon was consulted by the Armour family on the treatment of their daughter Lolita’s CDH.^9^ After thorough preparation, he operated on Lolita on both hips according to the Lorenz method on 31 December 1900: “Dr. Ridlon devoted months to a study of his little patient’s case before he operated on her, and did not apply his instruments until he had exhausted all the information which the surgical world had to offer on the partical disorder under consideration.”^10^

Two months later, on 23 February 1901, it was reported that “Dr. Ridlon’s operation upon Armours’s daughter proves successful,” after the girl had to lie in the plaster for two weeks. X-rays showed the success of the operation.^11^ After plaster removal on 8 May 1901, new X-rays confirmed that the “operation of several months ago on her hip bones was completely successful.” “The X-ray pictures show that she [Lolita] has been restored to health. She is able to go out for doors for recreation.”^12^

Thereafter, nothing can be read about Lolita in American newspapers. However, the results were unsatisfactory. Ridlon had operated on both hips; one femoral head had slipped out of the acetabulum again.^13^ The family was therefore looking for other options and doctors and came across Prof. Lorenz from Vienna. “A sorrowing mother in Chicago read of his ability and success.”^14^

Lorenz received a letter from the Armour family inviting him to come to Chicago to treat Lolita [12]. Her parents were afraid that a trip to Vienna for the operation would be too much of a burden for her daughter.

In July 1902, an Austrian newspaper reported that “Mrs. Ogden Armour from Chicago [ …] has arrived here to stay at the Sanatorium of the imperial counselor Dr. Rudinger at Purkersdorf [near Vienna] for a six-week course,”^15^ but she already left three weeks later.^16^ Only retrospectively one can guess that the meaning of Mrs. Armour’s stay in Vienna was to meet Adolf Lorenz and to negotiate his trip to Chicago.

In early October 1902, it was reported that famous surgeons were on their way to Chicago to operate Lolita Armour. “The case which calls him [Lorenz] to Chicago has baffled the skill of American surgeons.” “During the last year, the little girl has not experienced the ease and freedom of movement her parents hoped for. The child’s family at length decided to call in the foreign expert.”^17^^,^ 18^,^ 19^,^ 20

Lorenz and his assistant Dr. Müller arrived in Chicago via Genoa (sailed on 24 September 1902) and New York.^21^ In the run-up to Lorenz’s arrival, Chicago doctors announced that for the first time a European doctor had been called to Chicago.^22^ The fee was also addressed: “The price they asked, including their expenses over here, was $75,000.”^23^ However, it can be assumed that it was much higher—$100,000.^24^

Lorenz insisted that the surgeon of the first operation, John Ridlon, was involved in the current treatment.^25^

After all the instruments had arrived from Vienna,^26^ the operation itself began on 12 October 1902 at 11 a.m. with the administration of the anesthetics at Armour Residence, 3724 Michigan Avenue. At 11.30 a.m., the patient was placed in the operating chair. The surgeon Lorenz was assisted by Dr. Friedrich Müller, Dr. Dexter Ashley, Dr. Frank Billings, Dr. John Ridlon, and his assistant, Dr. J.L. Miller (Fig. 4).^27^

**Fig. 4:**
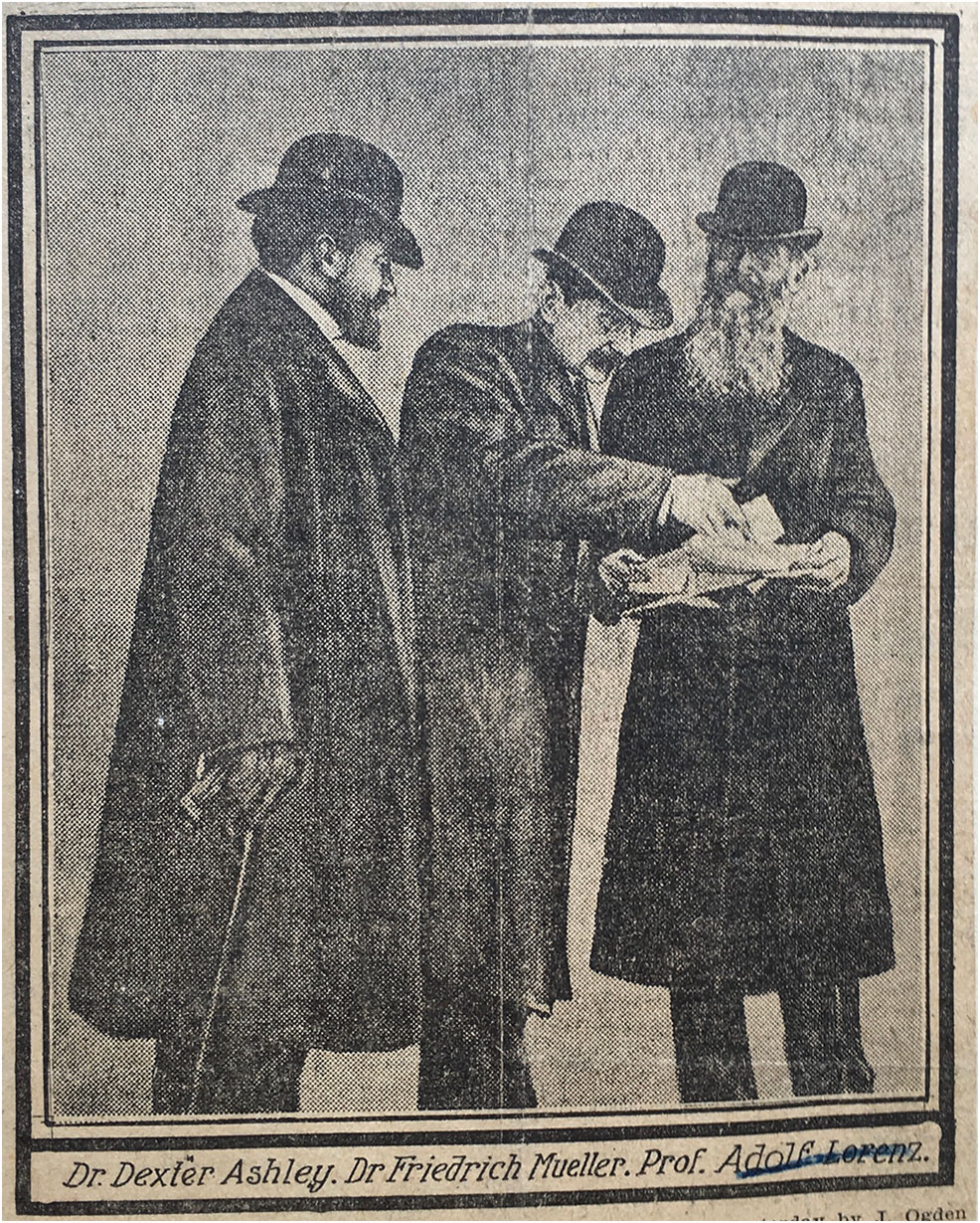
After surgery on October 12th 1902: Adolf Lorenz, Friedrich Müller, Dexter Ashley (Lorenz Archiv Vienna—Photograph: G. Holzer)

“It required about two hours to perform the operation. The right leg was drawn until the end of the femur reached the socket where it should rest. Then I turned the limb out at right angles and applied a heavy dressing. Over this was placed a thick plaster of paris cast, which will hold it in place until spring. The cast is so placed that it will allow free movement of the knee. So, it will be possible for the little girl to walk after two or three weeks,”Lorenz was quoted.^28^

“There is no doubt in my mind that the operation will be a success,” said Prof. Lorenz last night. “Of course, this will be proven, when the cast is removed.”^29^ In the first few days after the operation, Lorenz saw his patient several times.^30^

It was also mentioned that “after the operation Lorenz was taken to a spin in an automobile by J. Ogden Armour.”^31^

“Local physicians who have manifested great interest in the operation (…) will have several opportunities to observe Prof. Lorenz’s methods while he is here.”^32^ In addition to Lolita, Lorenz treated many patients who waited in bulk. Until 18 October, there were numerous clinics in various hospitals where he demonstrated the operation on poor children free of charge.^33^^,^ 34

But he also had enough paying patients. These consulted him about John Ridlon’s ordination^35^ or in his hotel. Hotel employees estimate the number of those at 200 to 300 people per day.^36^

In honour of Lorenz guest dinners and banquets were organized: On 13th October, he was a guest at Charles S. Bacon, 420 Center Street, “whose wife is a relative of the eminent surgeon” [1], and on 14 October, there was a Chicago Orthopedic Society banquet at the Chicago Athletic Club.^37^

The New York Times’ “Topic of the Times” commented about American national pride, and the contributions and developments of American medicine compared with abroad. “Until one studies the episode carefully it is distinctly humiliating to our National pride.” However, the successes of American medicine are so numerous and successful that American doctors “can well afford to accept instructions from the specialist of other lands.”^38^

Some doctors criticized Lorenz for treating patients without a license from the state of Illinois. Therefore, according to Frank Billings, “in order to prevent unpleasant complications, the Chicago doctors on the State Examination Commission granted Prof. Lorenz the right to immediate treatment of the sick.”^39^

“The case of Lolita Armour of Chicago, Professor Lorenz said, would not require further attention until spring. Then he will either return to this country or the child will be taken to Vienna.^40^

The evening before his departure from Chicago on 2 December 1902, Lorenz was given a dinner with Chicago colleagues in his honour.^41^ Because of the Armour family and other patients paying fees “Lorenz left the city a rich man.”^42^

From Chicago, Lorenz left for Washington, where he was received by the President of the USA, Theodore Roosevelt Jr.^43^^,^ 44 After having attended a theatre performance in New York on 30 December 1902, Lorenz boarded the White Star liner “Celtic,” which sailed to Europe on the morning of 31 December.^45^

It was estimated that while in this country Dr. Lorenz had performed almost 150 operations and his assistant, Dr. Müller, more than 25.^46^^,^ 47

After almost four months of absence, Lorenz returned to Vienna on 20 January 1903.^48^ Lorenz reported on his experiences in America in a public lecture on 2 March 1903.^49^

Already on 15 April 1903, Lorenz reached Chicago again to see Lolita Armour and to remove the plaster cast from her hip.^50^ “Every indication points to a successful outcome of Prof. Lorenz’s operation…” Lorenz said: “I found the skin in healthy condition and the bone in correct position.” “She can walk with only a slight limp, although the parts affected are still weak from long disease.” “As she gains strength she will have better control of her leg.”^51^ Understandably, Lolita and her mother reacted very emotionally: “Oh! Oh! Mamma, see, I can walk.”^52^

On 7 July, Lorenz traveled back to Europe on board the Crown Prince Wilhelm.^53^

In May 1903, a convention of the American Surgical and Orthopedic Association took place in Washington. The main topic of the lectures and discussions were Lorenz treatments. “The statement was made that the operation … was not entirely successful.”^54^^,^ 55 The invitation from Lorenz to America by J. Ogden Armour was nevertheless a blessing for the many other children who were also able to be treated.^56^^,^ 57

At the meeting of the New York Academy of Medicine: Section on Orthopedics (April 1904), John Ridlon presented the paper of the evening: “A consideration of the ultimate results of the bloodless replacements of congenital dislocated hip.”^58^ After that, the results from various clinics and hospitals were presented and the pros and cons of the Lorenz method discussed [6].

Already during Lorenz’s last stay, it had been agreed with the family that Lolita should come to Vienna to train and improve her surgical results. “He thought the trip might be necessary to teach her the art of walking and to give her limb perfect mobility,” said Mrs. Armour.^59^^,^ 60

Therefore, the Armours traveled to Europe, arrived in Vienna on 22nd April and moved into a suite at the Hotel Bristol.^61^ The next day, Lolita was examined by Prof. Lorenz: “She has made capital progress since the operation. The case is thoroughly satisfactory. After a short course of massage and manipulation of the injured joint the girl would be able to walk perfectly.”^62^^,^ 63

Lolita, her mother, and entourage stayed in Vienna all summer to receive intensive physical therapy, personally supervised by Lorenz (Fig. 5). They also spent some time in Lorenz’ Villa in Altenberg in Lower Austria [15]. It was only on 9 December 1904 that they returned to Chicago.^64^

**Fig. 5:**
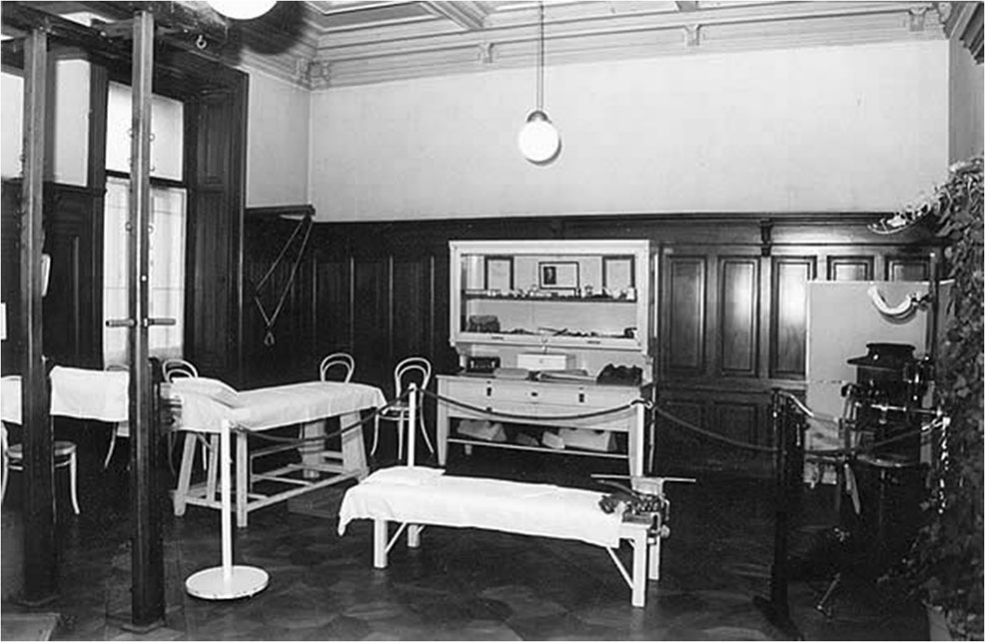
Gymnastics hall in Lorenz’ historical office in Vienna (Photograph: G. Holzer)

Thus, the treatment of Lolita by Prof. Lorenz was completed in autumn 1904. There is no final statement from Lorenz himself. Lolita’s mother reported on the treatment success after returning from Europe: “I am glad to say that Lolita has greatly improved. She can now walk and run about like other children. Dr. Lorenz’ s treatment has done wonders for her. She has been seven months under his constant care.”^65^ “Lolita was cured and the treatment was now at an end. She walks with a slight limp, but this will pass away in a few years.”^66^ “Lolita can walk miles at a time, and, in fact, part of her treatment in Vienna was to walk five miles a day.”^67^^,^ 68

As a perfect conclusion to the whole treatment process and media reports, the “New York Times” found a longer comment on the case:^69^ The parents’ optimism about the outcome of the operation by Lorenz cannot be shared by the medical community. It was probably true that Lolita benefited from the systematic exercises for the muscles. The parents subjectively saw this as an almost perfect result. The experts did not agree with this opinion. There was only a perfect result in just under 10%. Together with 50% anterior transformations, one can expect a satisfactory result from the “Lorenz Method” in about 60% of the treated hips. The best result of Lorenz’s visits in 1902 and 1903 was the attention to orthopaedic problems among children in America. “Miracles,” as they had been partly attributed to him by the press, could not have been hoped for by him.

## Footnotes

1 Armour, Philip Danforth Wikipedia https://en.wikipedia.org/wiki/Philip_Danforth_Armour (last accessed: 4th May 2020)

2 Armour, J. Ogden Wikipedia https://en.wikipedia.org/wiki/J._Ogden_Armour (last accessed 4th May 2020)

3 Wiener Klinische Rundschau 1896

4 McClure’s Magazine Jan 1903, Vol. XX, No. 3.

5 Wiener Medizinische Wochenschrift 1900, 23, pp. 1146

6 Medical Times and Register 8 Mar 8 1890, 62, 10, pp 293

7 Medical News 30 Apr 1898, 72, 18, pp. 570

8 Medical Times and Register Oct. 1899, 37, 10, pp 352

9 Chicago Daily Tribune (CDT) 13 Oct 1902

10 NYT 1 Jan 1901

11 NYT 23 Feb 1901

12 NYT 9 May 1901

13 McClure’s Magazine, Jan 1903, Vol. XX, No. 3.

14 Frank Leslie’s Popular Monthly, Jan 1903

15 Sport and Salon, 5 July 1902, p. 10

16 Sport und Salon, 26 July 1902, p. ??

17 CDT 5 Oct 1902

18 NYT 6 Oct 1902

19 CDT 11 Oct 1902

20 NYT 12 Oct 1902

21 Neues Wiener Tagblatt (NWT) 21 Jan 1903

22 CDT 5 Oct 1902

23 NYT 12 Oct 1902

24 McClure’s Magazine, Jan 1903, Vol. XX, No. 3.

25 McClure’s Magazine, Jan 1903, Vol. XX, No. 3.

26 NWT 21Jan 1903

27 CDT 13 Oct 1902

28 CDT 13 Oct 1902

29 CDT 13 Oct 1902

30 CDT 13 Oct 1902

31 CDT 13 Oct 1902

32 CDT 13 Oct 1902

33 NYT 14 Oct 1902

34 CDT 14 Oct 1902

35 McClure’s Magazine, Jan 1903, Vol. XX, No. 3.

36 Frank Leslie’s Popular Monthly, Jan 1903

37 CDT 14 Oct 1902

38 NYT 16 Oct 1902

39 Illustriertes Wiener Extrablatt, 22 October 1902

40 NYT 9 Dec. 1902

41 Deutsches Volksblatt 15 Dec. 1902

42 Frank Leslie’s Popular Monthly, Jan 1903

43 NY Tribune 30 Dec 1902

44 NWT 21 January 1903

45 NY Tribune 30 Dec 1902

46 NY Tribune 30 Dec 1902

47 NWT 21 Jan 1903

48 NWT 21 Jan 1903

49 NWT 27 Feb 1903

50 NYT 15 April 1903

51 CDT 17 April 1903

52 NYT 17 April 1903

53 NYT 27 June 1903

54 NYT 14 May 1903

55 CDT 14 May 1903

56 NYT 18 April 1903

57 NYT 27 Sept 1903

58 Medical News 16 April 1904

59 NYT 12 April 1904

60 Neue Freie Presse (NFP) 22 April 1904

61 NFP 22 April 1904

62 NWT 23 April 1904

63 NYT 24 April 1904

64 NYT 10 Dec 1904

65 NYT 4 Dec 1904

66 NYT 30 Nov 1904

67 NYT 10 Dec 1904

68 CDT 10 Dec 1904

69 NYT 11 Dec 1904
